# O5-8 Drive for Muscularity Behaviors in Male Bodybuilders: A Trans-Contextual Model of Motivation

**DOI:** 10.1093/eurpub/ckac094.040

**Published:** 2022-08-29

**Authors:** Stéphanie Meriaux-Scoffier, Lisa Chaba, Vanessa Lentillon-Kaestner, Fabienne d'Arripe-Longueville

**Affiliations:** 1 Université Côte d'Azur, LAMHESS, France; 2 University of Teacher Education of the State of Vaud, HEP-VD, Lausanne, Switzerland

**Keywords:** drive for muscularity behaviors, male bodybuilders, trans-contextual model of motivation, self-determination theory, theory of planned behavior

## Abstract

**Background:**

The drive for muscularity behaviors are very common in male athletes, especially in male bodybuilders. Studies have shown that drive for muscularity is a risk factor for eating disorders and muscle dysmorphia. Although several psychological variables have been related to drive for muscularity (e.g. body dissatisfaction), little is known about the motivational mechanisms underlying drive for muscularity.This study applied the trans-contextual model of motivation to the drive for muscularity behaviors of male bodybuilders at risk of developing muscle dysmorphia. The relationships between self-determination theory constructs and drive for muscularity behaviors, via the theory of planned behavior variables were examined.

**Methods:**

A total of 175 Swiss male bodybuilders (Mage = 27.34; *SD*age = 7.53) completed measures on motivation for sport, theory of planned behavior variables, and drive for muscularity behaviors. They practiced bodybuilding from three to 24 hours per week and had done so for 7.19 years on average. A series of path analyses was performed to determine the final model.

**Results:**

The fit indices of the final model were satisfactory: X^2^ (11) = 13.81; p = .244; TLI = .98; CFI = .99; RMSEA = .04. The model explained 29% of the variance of drive for muscularity behaviors. The final path analysis supported the motivational sequence, with autonomous motivation for sport showing a positive, significant and indirect association with the drive for muscularity behaviors via perceived behavioral control and intention to gain muscle mass, and controlled motivation for sport showing a positive association with the drive for muscularity behaviors both directly and via attitude and intention to gain muscle mass.

**Conclusions:**

It was concluded that the trans-contextual model of motivation applies only partially to the drive for muscularity behaviors in male bodybuilders. Preventive actions may be important for male bodybuilders, who focus on gaining muscle mass. Specifically, such actions should be directed toward helping them to avoid developing controlled motivation because, although motivation for sport of any kind can be a direct or indirect risk factor for the development of deviant behavior, controlled motivation seems be put them at greater risk.

